# Neurocognitive deficits in survivors of childhood acute myeloid leukemia

**DOI:** 10.1186/s12887-022-03369-0

**Published:** 2022-05-21

**Authors:** Satoko Takahashi, Satomi Sato, Shunji Igarashi, Hitoshi Dairoku, Yuichi Takiguchi, Tetsuya Takimoto

**Affiliations:** 1 Department of Pediatrics, Japanese Red Cross Narita Hospital, 90-1 Iida-cho, Narita-shi, Chiba, 286-8523 Japan; 2 Department of Medical Oncology, Chiba University Graduate School of Medicine, 1-8-1 Inohana, Chuo-ku, Chiba-shi, Chiba, 260-8677 Japan; 3 Graduate School of Public Health, St. Luke’s International University, OMURA Susumu & Mieko Memorial, St. Luke’s Center for Clinical Academia, 5th Floor, 3-6-2 Tsukiji, Chuo-ku, Tokyo, 104-0045 Japan; 4 Specified Nonprofit Corporation LD, Dyslexia Center, #315 Applaud Ichikawa, 3-1-1 Ichikawaminami, Ichikawa-shi, Chiba, 272-0033 Japan; 5 Children’s Cancer Center, National Center for Child Health and Development, 2-10-1 Okura, Setagaya-ku, Tokyo, 157-8535 Japan

**Keywords:** Acute myeloid leukemia, Neurocognitive deficits, Working memory, Childhood, Quality of life

## Abstract

**Background:**

Although treatment of acute myeloid leukemia (AML) contains neurotoxic agents, studies investigating neurocognitive outcomes in children with AML are sparse. We evaluated late cognitive effects in children treated with a high-dose cytarabine based regimen, focusing on general intellectual ability and specific neurocognitive domains.

**Methods:**

We evaluated 12 survivors of childhood AML who were treated between 2006 and 2016 and completed the Wechsler Intelligence Scales. One-sample *t*-tests were used to compare full-scale intelligence quotient (FSIQ) and primary index scores to norms. The overall effect of index scores and subtests was examined with one-way ANOVA. Univariate analyses and multiple regression models examined demographic and clinical characteristics associated with FSIQ.

**Results:**

Participants who underwent the Wechsler Intelligence Scale for Children demonstrated impairment on working memory index and participants who underwent the Wechsler Adult Intelligence Scale showed low score in the subtests that reflect working memory, whereas they exhibited no statistical differences versus the population means for FSIQ. There were no significant differences in the overall effect of index scores and subtests. On univariate analysis, FSIQ were related to time since diagnosis and age at assessment, and both were significant predictors of FSIQ on multiple linear regression.

**Conclusions:**

Survivors of childhood AML exhibited impairment of working memory, even if their FSIQ was within the normal range. Difficulties in specific cognitive domains are associated with reduced quality of life. It is important to identify survivors who are at risk and provide tailored interventions.

## Background

Acute myeloid leukemia (AML) accounts for 25% of all cases of childhood leukemia, and it affects approximately 150–200 patients annually in Japan [1]. Because of improvements in treatment and supportive care over time, event-free survival and overall survival rates for children with AML have approached 60% and 70%, respectively [1, 2]. These improvements have led to an increased focus on late effects in long-term survivors [3, 4]. Recently, late neurocognitive effects among cancer survivors have attracted increasing attention because neurocognitive problems may limit the quality of life and functional outcomes of patients [5].

Although the majority of the literature has focused on evaluating the effects of methotrexate on cognitive outcomes in survivors of childhood acute lymphoblastic leukemia (ALL) [5, 6], many chemotherapeutic agents may cause neurocognitive impairment. Several reports described acute cerebral and cerebellar toxicity following treatment with high-dose cytarabine (HD Ara-C) [7–10], which is used as a standard regimen for AML.

Because chemotherapy for childhood AML also contains neurotoxic agents and intrathecal therapy (IT) [1, 3], neurocognitive sequelae may occur in AML survivors. Nevertheless, few studies have examined neurocognitive outcomes associated with childhood AML.

This study evaluated the development of late cognitive effects following AML treatment in children with a focus on general intellectual ability and specific neurocognitive domains. In addition, we explored the associations of neurocognitive performance with clinical and demographic factors.

## Methods

### Participants

The inclusion criteria for this cross-sectional study were as follows: (1) diagnosis of AML or acute undifferentiated leukemia (AUL); (2) < 20 years of age at diagnosis; (3) ≥ 1 year off treatment during a continuous first remission; and (4) receipt of chemotherapy with HD Ara-C. Survivors were excluded if they had been diagnosed with a specific neurodevelopmental disorder such as Down syndrome, if their primary language was not Japanese, or if they had developed any relapse or second malignancy prior to neurocognitive testing. Survivors who underwent allogeneic hematopoietic stem cell transplantation (HSCT) were not excluded. The Institutional Review Board of the Japanese Red Cross Narita Hospital approved this study (Approval Number: 526–01). Informed consent for participation and medical record release was obtained from survivors or their proxies (for participants younger than 20 years), and assent was obtained from survivors where appropriate.

### Treatment protocol

In this study, patients with AML were treated with regimens primarily used in the AML-05 or AML-12 trials, both of which were nationwide multicenter studies conducted by the Japanese Pediatric Leukemia/Lymphoma Study Group. The treatment protocols in these trials consisted of two courses of induction therapy and three to four courses of intensification therapy with cytarabine, etoposide, mitoxantrone, idarubicin, and an age-adjusted dose of triple intrathecal therapy. Central nervous system (CNS) disease was defined as either ≥ 5 white blood cells with blasts in cerebrospinal fluid (CSF) or with clinical and radiographic signs of CNS leukemia. Patients with CNS disease received additional weekly IT until the CSF was clear of blasts. Cranial radiation therapy was not included in the protocols. Allogeneic HSCT was limited to the high-risk group [11–13]. The cumulative doses of cytarabine, anthracyclines, and etoposide in the AML-05 and AML-12 trials are noted in Table 1. Japanese trials used a higher cumulative dose of cytarabine than major studies in other countries [11].

**Table 1:** Cumulative doses of cytotoxic agents in the AML-05 and AML-12 studies

	**AML-05**		**AML-12**	
	LR	IR (HR)	LR; A/B	IR (HR); A/B
Cytarabine (g/m^2^)	77.4	77.4	78.4/95	77.4/94
Anthracycline equivalent (total, mg/m^2^)^a^	225	375	300	375
Mitoxantrone	25	55	40	55
Idarubicin	20	20	20	20
Etoposide (mg/m^2^)	1750	1750	2200	1750

*LR* low-risk, *IR* intermediate-risk, *HR* high-risk, A: ECM, B: HD-ECM

AML-12 used two regimens for induction therapy. ECM consisted of cytarabine, mitoxantrone, etoposide, and triple intrathecal therapy. HD-ECM consisted of high-dose cytarabine, mitoxantrone, etoposide, and triple intrathecal therapy

^a^The cumulative anthracycline dose was calculated relative to the amount of daunorubicin using a conversion rate of 5:1 for daunorubicin to mitoxantrone/idarubicin

### Neurocognitive testing

All participants completed an age-appropriate Japanese version of the Wechsler Intelligence Scales to assess general intelligence: the Wechsler Preschool and Primary Scale of Intelligence-Third Edition (WPPSI-III) for children younger than 5 years, the Wechsler Intelligence Scale for Children-Fourth Edition (WISC-IV) for children aged 5–16 years, and the Wechsler Adult Intelligence Scale-Fourth Edition (WAIS-IV) for survivors aged 17 years or older. One participant was tested using the WPPSI-III, seven patients were tested using the WISC-IV, and four patients were tested using the WAIS-IV. The WISC-IV and WAIS-IV include the full-scale intelligence quotient (FSIQ), verbal comprehension index (VCI), perceptual reasoning index (PRI), working memory index (WMI), and processing speed index (PSI). The WPPSI-III includes FSIQ, VCI, PRI, PSI, and a general language composite (GLC), whereas PSI and GLC are extrapolated from supplemental subtests. FSIQ and each index score have a standardized mean (M) and standard deviation (SD) of 100 and 15, respectively. The test was administered by one experienced psychologist under the supervision of another licensed psychologist.

### Statistical analysis

Descriptive statistics were calculated according to the clinical and demographic characteristics of the participants, including gender, age at diagnosis, time since diagnosis, history of HSCT, age at assessment, parental educational history, and household income.

A one-sample *t*-test was used to compare FSIQ and primary index scores (VCI, PRI, WMI, and PSI) to standard scores (M = 100, SD = 15). The WISC-IV and WAIS-IV scores displayed comparability if all common subtests are performed [14]; however, study participants were only tested for core subtests. For this reason, we separately examined primary indices obtained by a group of participants who underwent the WISC-IV or WAIS-IV. Because only one participant was tested with the WPPSI-III and PSI and GLC were not evaluated, we excluded that subject from the analysis of the primary indices. The overall effect of index scores and subtests was tested by one-way ANOVA. Univariate analyses (Fisher’s exact test, *t*-test, Pearson’s correlation, and Spearman’s correlation) examined demographic and clinical characteristics associated with FSIQ. Demographic and clinical variables identified as statistically significant (*p* < 0.05) on univariate analysis were included in multiple regression models. All statistical analyses were performed using EZR for R, a modified version of R commander designed to add statistical functions frequently used in biostatistics [15].

## Results

**Fig. 1:**
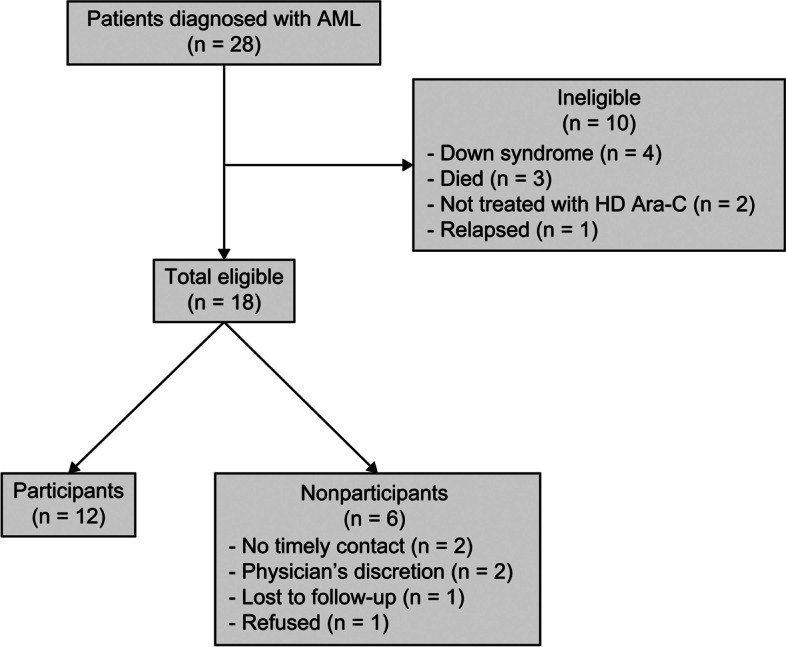
Patient selection

Twenty-eight patients < 20 years of age received a diagnosis of AML (*n* = 26) or AUL (*n* = 2) from 2006 to 2016. Among them, 18 patients satisfied the eligibility criteria for this study, and neurocognitive data were obtained from 12 patients. The reasons for exclusion or a lack of participation are noted in Fig. 1. On average, participants were 8.0 years old at diagnosis and 12.9 years old at assessment. Of the 12 participants, five received HSCT, three received total-body irradiation, and one presented with CNS disease at diagnosis. Participant with CNS disease showed ≥ 5 white blood cells with blasts in the CSF but without clinical CNS symptoms. Although brain magnetic resonance imaging performed in all patients before the treatment and an intracranial mass was detected in one patient, this patient was regarded CNS-negative because lack of clinical CNS symptoms. None of the participants developed neurological events or toxicity that affected other nervous systems during chemotherapy. One patient had a history of epilepsy, and none of participants had family medical history, such as psychiatric illnesses and epilepsy. Parental education and household income were used as proxies for family socioeconomic status. The full demographic characteristics of the participants are noted in Table 2.

**Table 2:** Demographic and clinical characteristics of survivors

	No
Total	12
Sex	
Male	5
Female	7
Mean age at diagnosis ± SD (range)	8.0 ± 5.4 (0.4–14.8)
Mean time from diagnosis ± SD (range)	5.2 ± 3.0 (2.3–11.1)
Prior CNS involvement	1
Positive history of HSCT	5
Mean age at assessment ± SD (range)	12.9 ± 6.0 (4.1–24.1)
Parental education	
High school	2
Vocational school/junior college	8
University	1
Unknown	1
Household income (Japanese yen)^a^	
< 6,000,000	6
≥ 6,000,000	4
Unknown	2

^a^6,000,000 Japanese yen is approximately 54,000 US dollars

SD, standard deviation; CNS, central nervous system; HSCT, hematopoietic stem cell transplantation

The participants’ index scores on the Wechsler Intelligence Scales in relation to normative data are summarized in Table 3. The WISC-IV and WAIS-IV were completed by seven and four participants, respectively. Participants who completed the WISC-IV displayed significant impairment on the WMI (*t* (6) =  − 3.36, *p* = 0.015, M = 83.3 [95% confidence interval = 71.1–95.4]) but no statistical difference from the normative M for FSIQ and other index scores. Among the participants who underwent the WAIS-IV, FSIQ and the four primary index scores did not differ significantly from the normative M of 100.

**Table 3:** Neurocognitive outcomes

	N	Mean	SD	Range	P*
FSIQ	12	97.5	12.1	81–123	0.488
FSIQ (WISC-IV)	7	93.7	9.30	81–104	0.124
FSIQ (WAIS-IV)	4	105.0	15.8	87–123	0.571
VCI	12	100.3	15.7	75–121	0.943
VCI (WISC-IV)	7	99.3	15.8	76–115	0.908
VCI (WAIS-IV)	4	103.0	19.7	75–121	0.781
PRI	12	98.6	14.2	80–124	0.736
PRI (WISC-IV)	7	94.1	13.3	80–115	0.287
PRI (WAIS-IV)	4	105.5	16.6	85–124	0.555
WMI	11	90.8	17.2	63–117	0.108
WMI (WISC-IV)	7	83.3	13.2	63–103	0.0152
WMI (WAIS-IV)	4	104.0	16.7	82–117	0.665
PSI	11	102.0	14.7	81–127	0.662
PSI (WISC-IV)	7	101.0	14.8	81–118	0.864
PSI (WAIS-IV)	4	103.8	16.8	87–12	0.686

^*^ One-sample *t*-test. *P-*value for calculated difference between participants and normative means (M = 100, SD = 15). Results with *P* ≤ 0.05 are regarded as statistically significant

*FSIQ* full-scale intelligence quotient, *VCI* verbal comprehension index, *PRI* perceptual reasoning index, *WMI* working memory index, *PSI* processing speed index

**Fig. 2:**
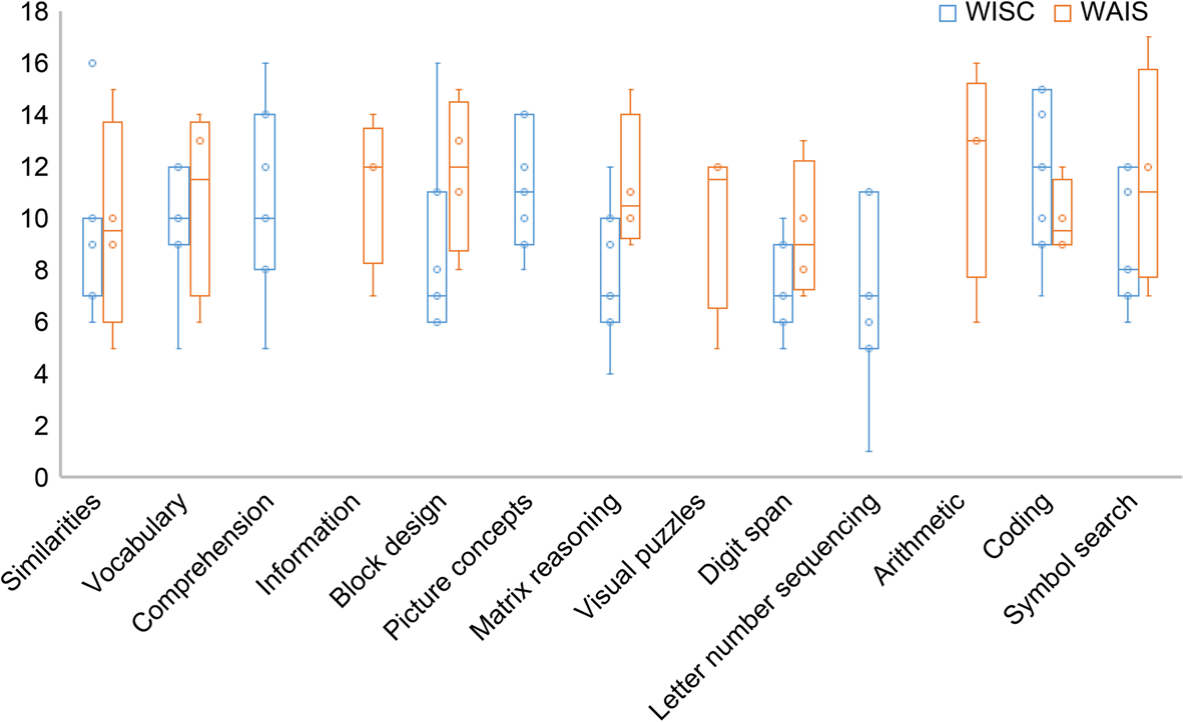
Wechsler Intelligence Scale for Children-Fourth Edition (WISC-IV) and Wechsler Adult Intelligence Scale-Fourth Edition (WAIS-IV) subtest scores. In the WISC-IV cases, the Letter-Number Sequencing subtest score was the lowest (mean [M] = 6.9, standard deviation [SD] = 3.2), and the Coding subtest score was the highest (M = 12.0, SD = 2.9). In the WAIS-IV cases, the Digit Span subtest score was the lowest (M = 9.5, SD = 2.3), and the Arithmetic subtest score was the highest (M = 12.0, SD = 3.7)

As presented in Fig. 2, among the subtest scores, the Letter-Number Sequencing subtest score was the lowest (M = 6.9, *SD* = 3.2) and the Coding subtest score was the highest (M = 12.0, SD = 2.9) in the WISC-IV cases, whereas the Digit Span subtest score was the lowest (M = 9.5, *SD* = 2.3) and the Arithmetic subtest score was the highest (M = 12.0, *SD* = 3.7) in the WAIS-IV cases. The Digit Span subtest score was also low in the WISC-IV cases (M = 7.4, SD = 1.8). However, neither group exhibited any significant differences in the overall effect of the index scores and subtests.

**Fig. 3:**
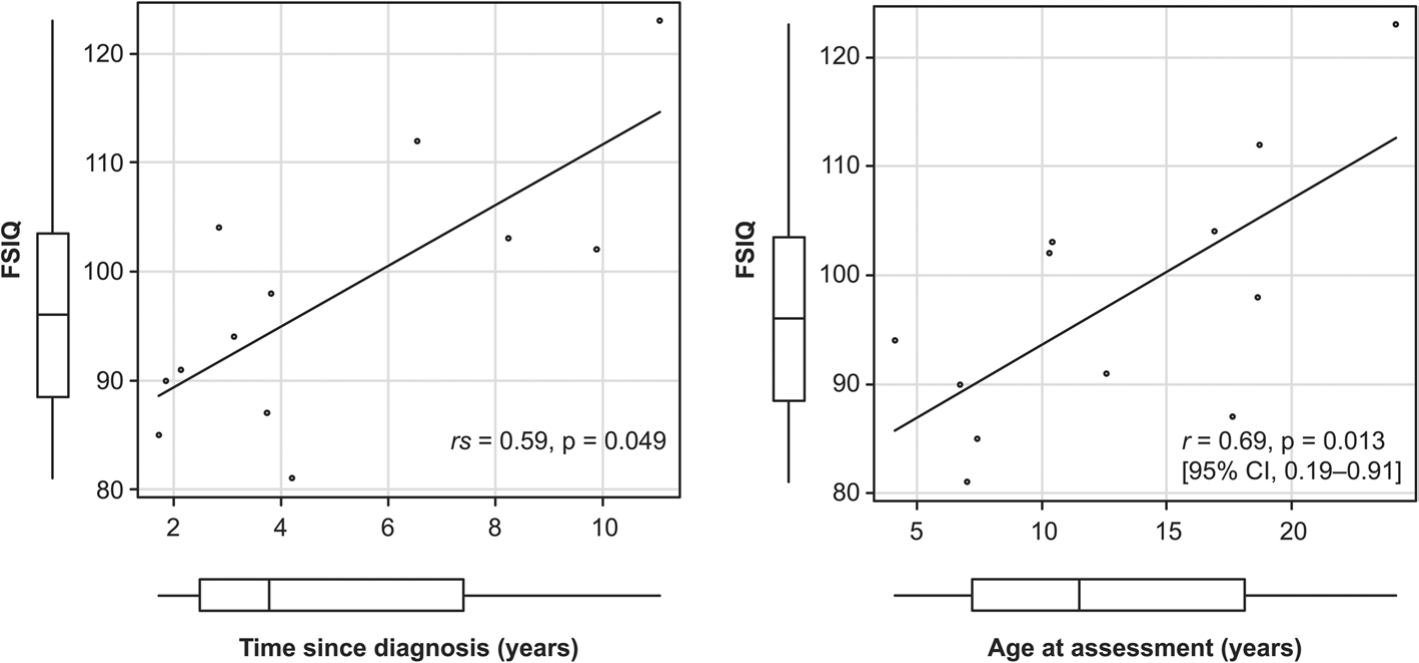
Associations of the full-scale intelligence quotient (FSIQ) with the time since diagnosis and age at assessment. Spearman's rank correlation suggested that FSIQ was moderately correlated with the time since diagnosis (*rs* = 0.59, *p* = 0.049). Pearson’s correlation coefficient exhibited a strong relationship between FSIQ and the age at assessment (*r* = 0.69, *p* = 0.013)

The associations between FSIQ and all demographic and clinical variables listed in Table 2 were examined by univariate analysis. The *t*-test revealed no significant difference in the FSIQ between the WISC-IV and WAIS-IV cases (M = 93.7, SD = 9.3 and M = 105.0, SD = 15.8, respectively, *p* = 0.16). Among WISC-IV cases, participants aged 10 years and older showed higher FSIQ score than participants under 10 years of age (M = 100.0, SD = 6.1 and M = 85.3, SD = 4.5, respectively, *p* = 0.017). As presented in Fig. 3, FSIQ was moderately related to the time since diagnosis (*rs* = 0.59, *p* = 0.049) and strongly related to age at assessment (*r* = 0.69, *p* = 0.013). A longer time since diagnosis and older age at assessment were associated with high FSIQ. Multiple linear regression was performed to predict FSIQ based on the time since diagnosis and age at assessment. A significant regression equation was found (*F* (2, 9) = 11.74, *R*^*2*^ = 0.66, *p* = 0.003). The participants’ predicted FSIQ was equal to 76.01 + 2.05 (time since diagnosis) + 0.88 (age at assessment), where time since diagnosis and age at assessment were measured in years. Both time since diagnosis and age at assessment were significant predictors of FSIQ.

## Discussion

The results of this study indicate that following treatment with HD Ara-C for AML, survivors exhibit impairment in working memory compared to population norms despite their comparable FSIQ. In multiple linear regression, we revealed that the time since diagnosis and age at assessment were both associated with FSIQ in survivors.

FSIQ did not differ between our AML survivor sample and Japanese population norms; however, deficits in working memory emerged among AML survivors. This is consistent with the findings of a recent report by the CCSS group, which suggested that survivors had a relative risk of impairment in at least one neurocognitive domain [16]. It was previously reported that deficits in specific cognitive domains may be unrelated to general intelligence [17]. In this study, participants who underwent the WISC-IV demonstrated significant impairment on the WMI. Participants performed poorly on the Digit Span subtest of the WAIS-IV, but impairment in the WMI score disappeared because of a high Arithmetic subtest score. Previous studies suggested that the Arithmetic subtest is a poor predictor of working memory ability, and the Digit Span and Letter-Number Sequencing subtests would be the strongest predictor variables [18, 19]. Because the Digit Span subtest more strongly reflects working memory than the Arithmetic subtest, working memory appears to be the most vulnerable function following childhood AML treatment.

Cerebellar dysfunction may lead to intellectual disorders, such as disturbances of executive function, including deficient planning, set-shifting, abstract reasoning, working memory, and decreased verbal fluency [20]. Cytarabine is known to cause cerebellar toxicity [7–10]. Autopsies demonstrated the loss of Purkinje cells in the cerebellum and reactive Bergmann glial cell proliferation in adults following HD Ara-C treatment [21]. Diffuse heterogeneous brain hypoperfusion, identified by single-photon emission computed tomography, has been reported in children with AML who received HD Ara-C [22]. Although we cannot state so categorically, it can be speculated that HD Ara-C treatment may cause late neurocognitive effects because of cerebellar toxicity. Prior research supported an association between chemotherapy for childhood ALL and long-term neurocognitive deficiencies [23]. Although IT in AML treatment is less frequent than in ALL treatment [12, 24]., similar neurocognitive sequelae may occur in AML since it contains same agents (methotrexate, cytarabine, and hydrocortisone) used in ALL treatment.

Several studies have described associations of poor neurocognitive outcomes following childhood cancer treatment with lower socioeconomic status, older age at diagnosis, cranial radiation therapy, and a history of seizures [25, 26]. In our study, a longer time since diagnosis and older age at assessment were significantly related to a higher FSIQ, whereas age at diagnosis, gender, parental education, and household income were not significantly associated with neurocognitive outcomes. The length of schooling or some curriculums might explain participants’ improvement of the weak cognitive domain; however, we could not investigate the relationship between neurocognitive performance and Japanese educational system in this study. The lack of a relationship between FSIQ and these variables could reflect our small sample size. Although some participants underwent HSCT with a total-body irradiation conditioning regimen (maximum, 12 Gray), no significant difference in FSIQ was found between participants who did and did not undergo HSCT. This result is consistent with recent findings suggesting that the risk of neurocognitive dysfunction does not differ between HSCT and chemotherapy-only treatment in patients with AML [16, 27]. We could not assess the impact of seizures on neurocognitive function, as only one survivor had a history of seizures in this study. This patient had pre-existing epilepsy but being without medication because of seizure-free for years.

This study had several limitations. First, the generalizability of the study findings may have been compromised by the small sample size, and our results need to be replicated in a larger sample. Second, the cross-sectional design of this study made it impossible to compare neurocognitive outcomes with baseline data. It is possible that participants in this study might have had an above-average FSIQ prior to diagnosis and that the average results observed in the participants might indicate a decline from a higher baseline level. Third, this study lacked a matched comparison group. Although we tried to use sibling controls, few participants had siblings who were close in age. Thus, we were limited to comparing the outcome to the standard Wechsler Intelligence Scale scores. Finally, although some genetic factors may contribute to individual vulnerability to chemotherapy [28–30], we did not examine any patients for genetic polymorphisms. Future studies, including an assessment of genetic polymorphisms, are required to fully understand the association between genetic factors and vulnerability to neurotoxic agents such as Ara-C.

The neurocognitive sequelae in children with AML have been considered more likely to have been caused by leukemia and its complications at presentation rather than by treatment [31]. By contrast, in our study, only one participant was CNS-positive at diagnosis, and no participants exhibited clinical CNS symptoms during treatment. Therefore, it is conceivable that the influence of the primary disease on cognitive function was small in this study.

## Conclusions

Participants displayed no impairment in FSIQ, but they had poor performance in working memory. Working memory is responsible for the temporary storage and manipulation of information. It affects all judgments and activities in everyday life, such as conversation, reading, writing, and calculation. Difficulties in specific cognitive domains, even with good general intellectual ability, may affect the quality of life of survivors of childhood AML. Individually administered intelligence test for survivors of childhood AML might be helpful in detecting domains of cognitive vulnerability. Early detection of neurocognitive late effects and development of interventions for survivors with neurocognitive deficit are required to provide survivors with optimal psychosocial care.

## Data Availability

The datasets generated during and/or analyzed during the current study are available from the corresponding author on reasonable request.

## Abbreviations

ALL: Acute lymphoblastic leukemia
AML: Acute myeloid leukemia
AUL: Acute undifferentiated leukemia
CNS: Central nervous system
CSF: Cerebrospinal fluid
FSIQ: Full-scale intelligence quotient
GLC: General language composite
HD Ara-C: High-dose cytarabine
HSCT: Hematopoietic stem cell transplantation
IT: Intrathecal therapy
M: Standardized mean
PRI: Perceptual reasoning index
PSI: Processing speed index
SD: Standard deviation
VCI: Verbal comprehension index
WAIS-IV: The Wechsler Adult Intelligence Scale-Fourth Edition
WISC-IV: The Wechsler Intelligence Scale for Children-Fourth Edition
WMI: Working memory index
WPPSI-III: The Wechsler Preschool and Primary Scale of Intelligence-Third Edition for children
